# Polar release of pathogenic Old World hantaviruses from renal tubular epithelial cells

**DOI:** 10.1186/1743-422X-9-299

**Published:** 2012-11-30

**Authors:** Ellen Krautkrämer, Maik J Lehmann, Vanessa Bollinger, Martin Zeier

**Affiliations:** 1 Department of Nephrology, University of Heidelberg, Im Neuenheimer Feld 162, 69120, Heidelberg, Germany; 2 Institute for Biology, Molecular Parasitology, Humboldt-UniversityBerlin, Berlin, Germany

**Keywords:** Hantavirus, Release, Polarized epithelia, Kidney, HFRS, MDCK

## Abstract

**Background:**

Epithelio- and endotheliotropic viruses often exert polarized entry and release that may be responsible for viral spread and dissemination. Hantaviruses, mostly rodent-borne members of the Bunyaviridae family infect epithelial and endothelial cells of different organs leading to organ dysfunction or even failure. Endothelial and renal epithelial cells belong to the target cells of Old World hantavirus. Therefore, we examined the release of hantaviruses in several renal epithelial cell culture models. We used Vero cells that are commonly used in hantavirus studies and primary human renal epithelial cells (HREpC). In addition, we analyzed MDCKII cells, an epithelial cell line of a dog kidney, which represents a widely accepted in vitro model of polarized monolayers for their permissiveness for hantavirus infection.

**Results:**

Vero C1008 and primary HREpCs were grown on porous-support filter inserts for polarization. Monolayers were infected with hantavirus Hantaan (HTNV) and Puumala (PUUV) virus. Supernatants from the apical and basolateral chamber of infected cells were analyzed for the presence of infectious particles by re-infection of Vero cells. Viral antigen and infectious particles of HTNV and PUUV were exclusively detected in supernatants collected from the apical chamber of infected Vero C1008 cells and HREpCs. MDCKII cells were permissive for hantavirus infection and polarized MDCKII cells released infectious hantaviral particles from the apical surface corresponding to the results of Vero and primary human epithelial cells.

**Conclusions:**

Pathogenic Old World hantaviruses are released from the apical surface of different polarized renal epithelial cells. We characterized MDCKII cells as a suitable polarized cell culture model for hantavirus infection studies.

## Background

The mechanism of viral spread often contributes to the clinical picture, for example because the process of egress and transport of viral particles to more distant target organs may determine the difference between local and systemic infection [1,2]. Polarized epithelia or endothelia facing the lumen represent the site of a pathogen attack. Due to the gate and fence function of polarized monolayers the infection of these cells requires specialized entry and egress strategies to overcome the barrier. The way of entry is mainly determined by the apical or basolateral localization of receptors on the surface of target cells. However, the budding from infected polarized cells requires the highly specific trafficking of viral components to the budding site. Many viruses exert side-specific entry and egress [3-5]. Hantaviruses, members of the Bunyaviridae family, infect epithelia and endothelia of different distant organs [6-12]. Viruses are transmitted to humans via inhalation of aerosols contaminated with excreta of infected rodents [13]. Upon inhalation hantavirus infects pulmonary and immune cells and during the clinical course, endothelia and epithelia of other organs may be targeted. Hantaviruses differ in the clinical picture regarding severity, and show a broad variability in the organ manifestation [14,15]. New World hantaviruses cause hantaviral cardiopulmonary syndrome (HCPS) and Old World hantaviruses cause hemorrhagic fever with renal syndrome (HFRS) or the milder form Nephropathia epidemica (NE). However, antigen of both viruses can be found in epithelia and endothelia of different organs and the mode of hantaviral spread is not well characterized so far.

The entry and release of the New World hantavirus Black Creek Canal virus (BCCV) has been described to occur preferentially at the apical surface of polarized Vero cells [16]. For the Old World hantaviruses HTNV and PUUV we could show that the viruses enter Vero and HUVE cells via the apical surface [17]. The mechanism of side-specific release of Old World hantaviruses from polarized cells has not been investigated thus far. Therefore, we examined the release of the pathogenic Old World hantaviruses PUUV and HTNV from pola-rized renal epithelial cells. We analyzed the release from polarized Vero C1008 cells and human primary renal tubular cells. Since MDCKII cells represent a well established and widely used model for polarized epithelial monolayers [18,19], we also investigated the susceptibility of MDCKII cells as a possible cell culture model for hantavirus infection and analyzed entry and release of hantaviruses in this cell line.

## Results

### Release of hantaviruses HTNV and PUUV from the apical surface of Vero C1008 cells and primary HREpCs

**Figure 1:**
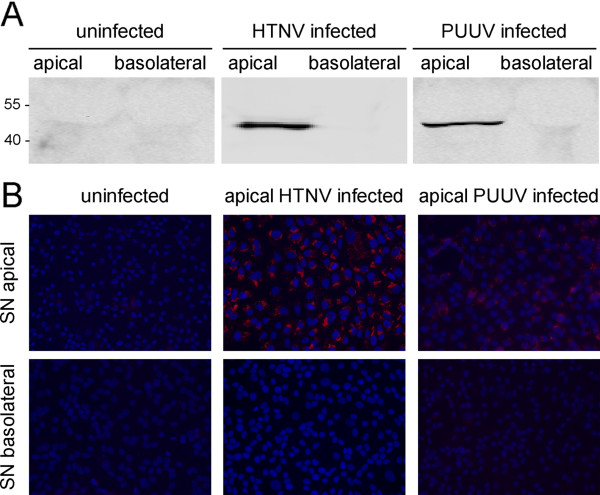
**Release of hantaviruses HTNV and PUUV from the apical surface of Vero C1008 cells. A**) Polarized Vero C1008 cells were infected with HTNV and PUUV via the apical surface and cell-free supernatants of apical and basolateral chambers were analyzed for the presence of hantavirus nucleocapsid protein by Western blot. **B**) Re-infection of Vero E6 cells with supernatants (SN) of polarized Vero C1008 cells infected with HTNV and PUUV. Cells were analyzed for the expression of nucleocapsid protein of HTNV and PUUV by immunofluorescence at day 4 and 8 post infection, respectively.

To examine the release of hantaviruses, Vero C1008 cells were cultivated on membrane inserts and infected with HTNV and PUUV by apical inoculation. Supernatants were collected from the apical and basolateral chamber and analyzed for the presence of hantaviral antigen at day 4 (HTNV) and at day 8 (PUUV) post infection. Hantaviral nucleocapsid protein was detected in cell-free supernatants from the apical but not the basolateral chamber (Figure 1A). The polar release of infectious virus was examined by incubation of Vero E6 cells (Figure 1B). At day 4 (HTNV) and at day 8 (PUUV) post infection, infected Vero E6 cells were only observed if cells were inoculated with supernatants collected from the apical chamber of infected cell cultures. Together, these results demonstrate that pathogenic Old World hantavirus HTNV and PUUV are released from the apical surface of Vero C1008 cells.

**Figure 2:**
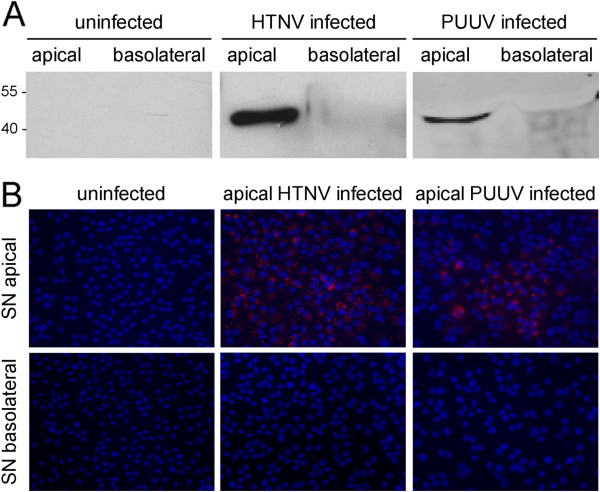
**Polar release of HTNV and PUUV from HREpCs. A**) Polarized HREpC were infected with HTNV and PUUV via the apical surface and cell-free supernatants of apical and basolateral chambers were analyzed for the presence of hantavirus nucleocapsid protein by Western blot. **B**) Re-infection of Vero E6 cells with supernatants of polarized HREpC infected with HTNV and PUUV. Infection was monitored by the detection of hantaviral nucleocapsid protein by immunofluorescence.

We wanted to verify our results in target cells relevant in human hantaviral infection. We infected polarized primary HREpC with HTNV and PUUV via the apical chamber and analyzed the cells at day 4 and 8 post infection, respectively. As for Vero C1008, particles were released from the apical surface as demonstrated by detection of hantaviral nucleocapsid protein by Western blot and re-infection of Vero E6 cells with supernatants from infected polarized HREpCs (Figure 2).

### HTNV infection of MDCKII cells

**Figure 3:**
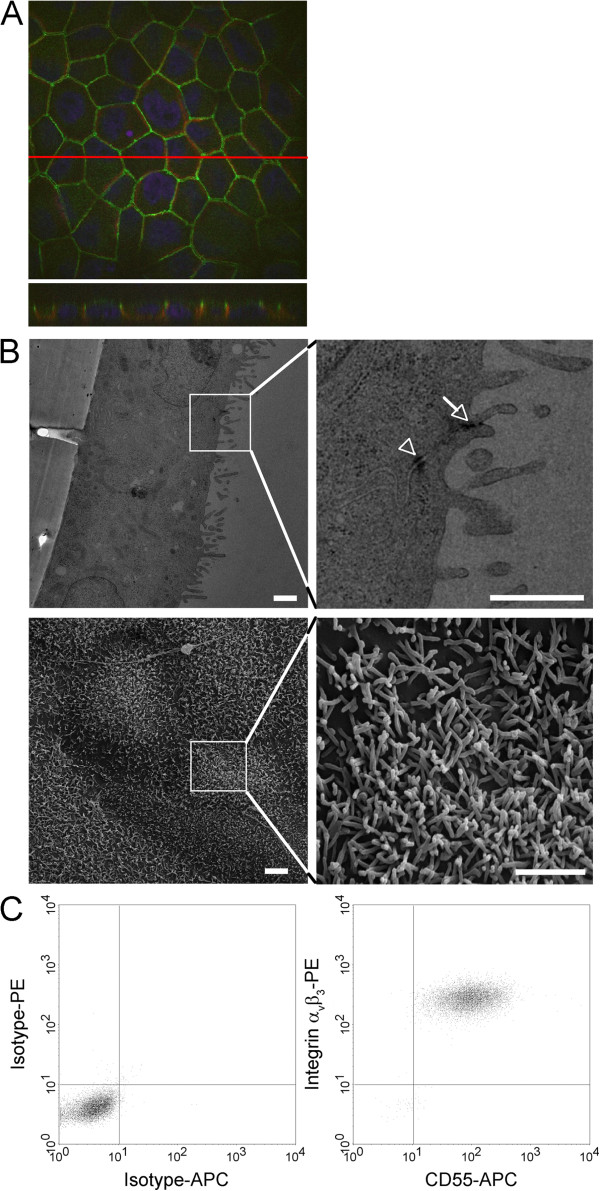
**Expression of hantavirus receptors CD55 and integrin α**_**V**_**β**_**3 **_**in MDCKII cells. A**) Polarized MDCKII cells were analyzed for the expression of ZO-1 (green) and β-catenin(red) and analyzed by confocal microscopy. Nuclei (blue) were stained with Hoechst 33342. **B**) Electron microscopy revealed polarization of MDCKII cells cultured on transwell filters. Upper panel: Transmission electron micrographs of polarized MDCKII cells exhibited cells growing a confluent monolayer with junctional complexes (tight junction and adherens junction, respectively indicated by white arrow and a desmosome indicated by arrowhead) at cell-to-cell contact sites. Lower panel: Scanning electron micrographs of MDCKII cells exhibited a confluent cell layer with the apical formation of a typical dense meshwork of microvilli. Scale bars: upper panel: 500 nm, lower panel 2 um. **C**) Surface expression of CD55 and integrin α_V_β_3_. MDCKII cells were stained with anti-CD55-APC antibody and anti-integrin α_V_β_3_-PE antibody and analyzed by flow cytometry.

MDCKII cells represent a well-established cell culture model for polarized epithelia. Therefore, we analyzed their potential use for hantavirus studies. Polarized MDCKII cells were analyzed by confocal immunofluo-rescence and electron microscopy (Figure 3). Z-sections of confocal image showed localization of ZO-1 and β-catenin at the intercellular junctions with ZO-1 being concentrated at the apical end of the cell-to-cell contacts. Cells exhibited a polar phenotype with tight junctions and microvilli at the apical surface. Hantaviral entry is mediated by integrin α_V_β_3_ and CD55 [17,20,21]. Flow cytometric analysis revealed that MDCKII cells express these receptors on their surface.

**Figure 4:**
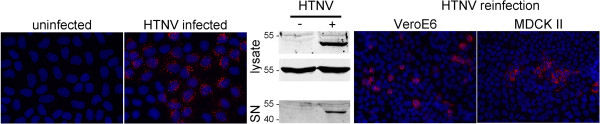
**Productive infection of MDCKII cells with HTNV.** MDCKII cells were infected with HTNV and analyzed for the presence of nucleocapsid protein by immunofluorescence and Western blot. Equal loading in cellular lysates uninfected and HTNV-infected cells was controlled by detection of tubulin. Release of particles was monitored by detection of nucleocapsid protein in cell-free supernatant of infected MDCKII cells. Release of infectious particles was assessed by re-infection of Vero E6 and MDCKII cells with supernatants of infected MDCKII cells and detection of nucleocapsid protein of HTNV by immunofluorescence.

**Figure 5:**
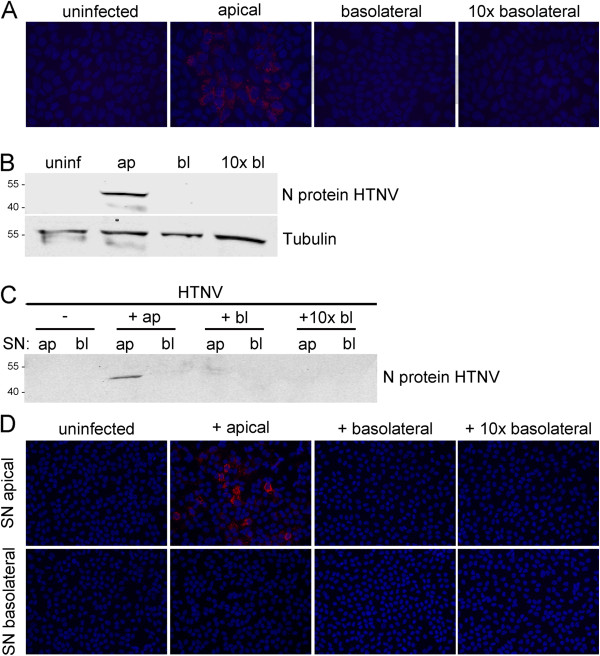
**Polar entry and release of HTNV from MDCKII cells. A**) Polarized MDCKII monolayers were infected with HTNV and analyzed for the expression of nucleocapsid protein by immunofluorescence. **B**) MDCKII cells were inoculated with HTNV via the apical or basolateral chamber. MDCKII cells were lysed and hantaviral nucleocapsid protein was detected by Western blot. Equal loading was monitored by detection of tubulin. **C**) MDCKII cells were inoculated with HTNV via the apical or basolateral chamber. Cell-free supernatants of the apical and basolateral chamber of infected polarized MDCKII monolayers were analyzed for the presence of nucleocapsid protein by Western blot. **D**) Apical and basolateral cell-free culture media of infected MDCKII cells were added to Vero E6 cells and re-infection was monitored by the detection of viral nucleocapsid protein.

Nonpolarized MDCKII cells were inoculated with HTNV and infection was monitored at day 4 post infection by detection of hantaviral nucleocapsid protein via immunofluorescence and Western blot (Figure 4). Cell-free supernatant was analyzed for the presence of nucleocapsid protein (day 4 post infection) and was added for re-infection to Vero E6 and MDCKII cells. Viral particles were released from MDCKII cells and were infectious for Vero E6 and MDCKII cells. In the next step we analyzed the entry and release of HTNV in polarized MDCKII cells. As demonstrated in Figure 5 MDCKII cells were infected via the apical side. Hantaviral nucleocapsid protein was detected at day 4 post infection in apically infected cells and its lysates by immunofluorescence and Western blot (Figure 5A, B). As for Vero C1008 and HREp cells we investigated the release of HTNV from MDCKII cells via detection of nucleocapsid protein in the supernatant and re-infection of Vero E6 cells. As demonstrated in Figure 5C and D, viral nucleocapsid protein was found in apical medium of infected cells. Infectious HTNV particles in the apical supernatant of infected polarized MDCKII cells re-infected Vero E6 cells.

### PUUV infection of MDCKII cells

**Figure 6:**
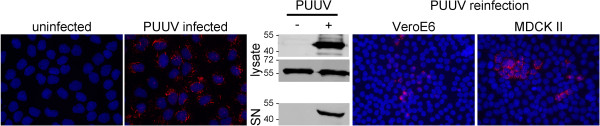
**Infection of MDCKII cells with PUUV.** MDCKII cells were grown on coverslips and infected with PUUV. Infection was analyzed by detection of nucleocapsid protein by immunofluorescence and Western blot. Equal loading in cellular lysates uninfected and HTNV-infected cells was controlled by detection of tubulin. Release of viral particles was analyzed by detection of nucleocapsid protein in cell-free supernatants of infected cells by Western blot and detection of viral nucleocapsid protein in Vero E6 and MDCKII cells re-infected with supernatants of infected MDCKII cells.

Entry and release of PUUV and HTNV do not differ in cells that have been used so far for hantavirus studies. Therefore, we wanted to know whether MDCKII cells are also permissive for infection with PUUV. Cells were incubated with PUUV and infection was detected at day 8 post infection by immunofluorescence and Western blot of cell lysate for PUUV nucleocapsid protein. Release of particles was demonstrated by the presence of hantaviral nucleocapsid protein in the supernatant of infected cells. This supernatant of infected cells was used to re-infect Vero E6 and MDCKII cells (Figure 6).

**Figure 7:**
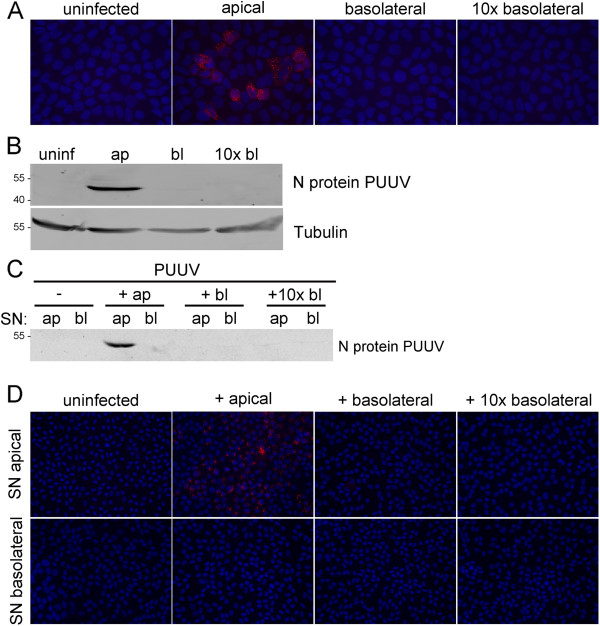
**Polar entry and release of PUUV from MDCKII cells. A**) Polarized MDCKII monolayers were infected with PUUV and hantaviral nucleocapsid protein was detected by immunofluorescence. **B**) Entry of PUUV in polarized MDCKII cells was analyzed by the detection of nucleocapsid protein in cell lysates by Western blot. **C**) MDCKII cells were inoculated with PUUV via the apical or basolateral chamber. Cell-free supernatants of infected polarized MDCKII cells were analyzed for the presence of PUUV nucleocapsid protein by Western blot. **D**) MDCKII cells were inoculated with PUUV via the apical or basolateral chamber. Supernatants of the apical and basolateral chamber of MDCKII cells were collected and added to Vero E6 cells. Re-infection of Vero E6 cells was analyzed by immunofluorescence of hantaviral nucleocapsid protein.

To analyze entry and release of PUUV in polarized MDCKII cells, cells were cultivated on cell culture inserts and inoculated with PUUV (Figure 7). Infection was monitored by immunofluorescence and Western blot analysis at day 8 post infection (Figure 7A and B). Nucleocapsid protein of PUUV was detected only in cells inoculated with virus via the apical chamber. The analysis of the medium of the apical and basolateral chambers of polarized MDCKII cells for PUUV nucleocapsid protein revealed the presence of nucleocapsid protein in supernatants from the apical chamber of infected cells (Figure 7C). Infectious particles were found in the supernatant of the apical chamber of apically infected MDCKII cells by incubation of Vero E6 cells (Figure 7D). These results demonstrate that entry and release of hantavirus PUUV occur at the apical surface of kidney cells corresponding to the mechanism of HTNV.

## Discussion

The mechanism of entry and release of viruses in polarized cells may play a key role in the pathogenesis. Entry often requires the crossing of epithelia with disruption of cell-to-contacts and the mode of release influences the dissemination of the pathogen causing local or systemic effects with consequences for the clinical picture and outcome of the disease [1,22,23]. In a previous study we could demonstrate the apical entry of Old World hantaviruses in epithelial and endothelial cells [17]. Here, we analyzed the egress of HTNV and PUUV and could demonstrate that the release of hantaviruses occurs from the apical surface of polarized epithelial cells. The cell surface site of entry and release corresponds to the mode of the New World hantavirus BCCV that also exhibit a predominant apical infection and egress, whereas epithelial cells bidirectionally release Andes virus (ANDV) [16,24]. Several differences in entry and assembly within the genus hantavirus have been described. During entry pathogenic and non-pathogenic hantaviruses exhibit differences in receptor usage and Old and New World hantaviruses vary in the interaction with cytoskeletal components [25-27]. Variations in entry and maturation were also observed within the group of Old World hantaviruses. In contrast to the New World hantaviruses Sin Nombre and Prospect Hill and the Old World hantaviruses Hantaan and Puumala virus, the newly identified Sangassou virus (SANGV) does not use CD55 as coreceptor for entry [17,20,21]. Old World hantaviruses have been described to mature in the Golgi. In contrast, particles of New World hantaviruses are reported to mature at the plasma membrane and characterization of the maturation of a newly described Old World hantavirus strain Hantaan HV114 suggests the ER-Golgi compartment and the plasma membrane as possible sites for virion assembly [16,28]. Therefore, the study of entry, maturation and release of different pathogenic and non-pathogenic hantaviruses will help to understand the underlying molecular mechanism of hantaviral disease.

The polarized release of viruses requires the trafficking and sorting machinery to route the viral proteins to the site of assembly and virions to the site of budding [29-31]. The coordinated process of sorting involves cellular proteins such as the cytoskeletal proteins, actin and tubulin, and GTPases [32,33]. Hantaviruses infect a broad spectrum of different cell types in vitro. Cells of human, primate and rodent origin are described to be susceptible for hantavirus infection [11,34,35]. Viruses also show differences in the mechanism and site of entry/release in different epithelial cells [36-39]. MDCK cells are a well established cell culture model to study trafficking and viral pathogenesis in epithelial cells [19,40,41]. Therefore, we analyzed the susceptibility and mechanisms of entry and release of hantaviruses in this cell line. MDCKII cells are permissive for hantavirus infection. Entry and egress occur apically and these results are consistent with the findings in Vero C1008 and human primary renal cells. However, the infection of polarized MDCKII is less efficient than the infection of VeroC1008 or HREpCs. Together, apical entry and release of Old World hantaviruses may play a role in the pathogenesis of hantaviral disease and the investigation of virus assembly and egress in a suitable cell culture model will allow the identification of viral and cellular determinants that are required for the production of infectious particles in polarized cells. Therefore, further studies will focus on the assembly of hantaviral proteins into virions in epithelial cells to further analyze the mechanism of polarized release that may be responsible for viral spread and that may have impact on the clinical picture.

## Conclusions

Pathogenic Old World hantaviruses HTNV and PUUV are released from the apical surface of polarized epithelial cells of the kidney. The apical site of infection and release was demonstrated in primary HREpCs and Vero C1008 cells. Furthermore, MDCKII cells were identified as suitable cell culture model for hantavirus infection studies. Corresponding to the results in Vero C1008 and HREp cells, hantaviral entry and release in MDCKII cells occur at the apical site. The use of MDCKII cells that represent a well established model for studying processes and interactions in polar epithelia displays a new helpful tool to investigate steps of the hantaviral replication cycle.

## Methods

### Cells

Vero C1008, Vero E6 (both kindly provided by G. Darai, Heidelberg) and Madin-Darby canine kidney II (MDCKII) (European Collection of Cell Cultures, ECACC) cells were maintained in Dulbecco’s modified Eagle’s medium supplemented with 10% fetal calf serum and antibiotics. Human renal proximal epithelial cells, HREpC, were obtained from Promocell and maintained in renal epithelial cell growth medium 2 (Promocell). Only HREpCs from passage two to six were used. For polarized monolayers, cells (1×10^5^) were plated on 0.4-μm-pore-size 12 well cell culture inserts (Greiner Bio-One) and cultivated for 7 days.

### Virus and infection

The stocks of hantavirus species Hantaan virus, strain 76–118 (HTNV) (kindly provided by G. Darai, Heidelberg) or Puumala virus, strain Vranica (PUUV) (kindly provided by S. Essbauer, Munich) were propagated on Vero E6 cells. To infect cells, virus inocula, HTNV or PUUV, at an MOI of 0.01 were added to Vero C1008, HREpC, or MDCKII cells; after incubation for 1 h at 37°C, unbound virus was removed by a triple washing. Cells were grown in tissue culture dishes containing coverslips. The infection was monitored by the immunofluorescence or by the Western blot analysis of hantaviral nucleocapsid protein expression. Expression of nucleocapsid protein of HTNV and of PUUV was analyzed in all cell types at day 4 and day 8 post infection, respectively. An equal loading was verified by the detection of tubulin on the same membrane. For re-infection, cells grown on coverslips were inoculated with cell-free supernatants of infected cells and monitored for infection.

### Immunofluorescence and Western blot analysis

For immunofluorescence, cells grown on coverslips or on cell culture inserts were actone-fixed and stained with primary and appropriate fluorescently labelled secondary antibodies. The following antibodies were used: mouse monoclonal anti-nucleocapsid protein (Progen), rabbit anti-ZO-1 (Invitrogen), mouse anti-β-catenin (Santa Cruz). Images were taken using a Nikon DXM1200C camera attached to a Nikon Eclipse 80i upright microscope (Nikon). Series of optical sections distanced 0.15 μm on the z axis were taken with a Perkin Elmer spinning disc confocal ERS-FRET on Nikon TE2000 inverted microscope.

For Western blot analysis, cells were lysed and after being boiled in SDS sample buffer and separated by SDS-PAGE, transferred to a nitrocellulose membrane. The Western blot analysis was performed after the incubation with primary antibodies by using near infrared fluorescent dye (IRDye)-conjugated secondary antibody and an Odyssey infrared imaging system (Li-Cor Biosciences). The following primary antibodies were used: rabbit polyclonal anti-PUUV or anti-HTNV nucleocapsid protein antibody and mouse anti-α-tubulin DM 1A (Sigma).

### Electron microscopy

For analysis by scanning electron microscopy (SEM), MDCKII cells were fixed with 2.5% (v/v) glutaraldehyde and 2% (w/v) paraformaldehyde in 100 mM cacodylate buffer (pH 7.4) for 30 min at room temperature. After fixation cells were rinsed three times for 10 min with 100 mM cacodylate buffer, and dehydrated through a graded ethanol series. After washing three times with hexamethyldisilazane (Electron Microscopy Sciences) cells were coated with gold and analyzed on a LEO 1430 scanning electron microscope.

For analysis by transmission electron microscopy (TEM), MDCKII cells were fixed with 2.5% (v/v) glutaraldehyde and 2% (w/v) paraformaldehyde in 100 mM cacodylate buffer (pH 7.4) for 30 min. Cells were rinsed three times for 5 min with 100 mM cacodylate buffer, postfixed for 1 h in 1% (v/v) osmiumtetroxide, rinsed three times with distilled water, *en bloc* stained with 0.5% (v/v) uranyl acetate, dehydrated through a graded ethanol series and finally embedded using EMBed 812 (Electron Microscopy Sciences). Cells were cut perpendicular to the substrate and 70–90 nm sections were collected. Sections were counterstained with 4% (w/v) uranyl acetate followed by lead citrate. All samples were imaged on a transmission electron microscope equipped with a wide-angle CCD camera (Zeiss EM 900, TRS Systems).

### Flow cytometry

For flow cytometry MDCKII cells were washed, scraped and stained with allophycocyanin (APC)-conjugated rabbit polyclonal anti-CD55 antibody, clone IA10 (BD Pharmingen) and phycoerythrin (PE)-conjugated mouse anti-integrin α_V_β_3_antibody, clone LM609 (Millipore). After 1 hour of incubation the cells were washed and then analyzed by flow cytometry with FACSCalibur (BD Pharmingen). Controls were incubated with APC- and PE-conjugated mouse and rabbit isotype antibodies.

## Competing interests

The authors declare that they have no competing interests.

## Authors’ contributions

EK designed the experiments and wrote the manuscript. MJL performed electron microscopy of polarized cells and contributed to the manuscript. VB performed the experiments. MZ was involved in all experiments and revised the manuscript. All authors read and approved the manuscript.
